# Plant community associations of two invasive thistles

**DOI:** 10.1093/aobpla/plv065

**Published:** 2015-06-02

**Authors:** Emily S.J. Rauschert, Katriona Shea, Sarah Goslee

**Affiliations:** 1 Department of Biological, Geological and Environmental Sciences, Cleveland State University, Cleveland, OH 44115, USA; 2 IGDP in Ecology and Department of Biology, The Pennsylvania State University, University Park, PA 16802, USA; 3 USDA-ARS, Pasture Systems and Watershed Management Research Unit, University Park, PA 16802, USA

**Keywords:** *Carduus*, community dynamics, co-occurrence, invasive species, plant invasions

## Abstract

In order to combat the growing problems associated with biological invasions, many researchers have focused on identifying which communities are most vulnerable to invasion by exotic species. However, once established, invasive species can significantly change the composition of the communities that they invade. The first step to disentangling the direction of causality is to discern whether a relationship with other vegetation exists at all. *Carduus nutans* and *C. acanthoides* are similar invasive thistles, which have caused substantial economic damage worldwide. We assessed the associations between the thistles and the standing flora in four sites in central Pennsylvania in which they co-occur. After sampling nearly 2000 plots of 1 m^2^, we used partial Mantel tests to assess the differences in vegetation between thistle and non-thistle plots after accounting for location, and non-metric multidimensional scaling to visualize differences among plots and sites. We found significant differences in community composition in plots with and without *Carduus* thistles. The non-native species *Sisymbrium officinale* and *Coronilla varia* were consistently associated with the presence of *Carduus* thistles. Several species were associated with areas that were free of *Carduus* thistles, including an important non-native pasture species (*Trifolium repens*). We found no evidence for differences in composition between plots with *C. nutans* versus *C. acanthoides*, suggesting that they have similar associations with the vegetation community. We conclude that even at the within-field scale, areas invaded by *Carduus* thistles have different vegetation associations than uninvaded areas, allowing us to target future research about the role of vegetation structure in resisting and responding to invasion.

## Abstract (precis)

We assessed the field-scale plant community associations of *Carduus nutans* and *C. acanthoides*, two similar, economically important invasive thistles. Several plant species were associated with the presence of *Carduus* thistles while others, including an important pasture species, were associated with *Carduus* free areas. Thus, even within fields, areas invaded by *Carduus* thistles have different vegetation than uninvaded areas, either because some plants can resist invasion or because invasion changes the local plant community. Our results will allow us to target future research about the role of vegetation structure in resisting and responding to invasion.

## Introduction

In order to combat the growing problems associated with biological invasions, there has been a focus on identifying which communities are most vulnerable to invasion by exotic species (Baker 1974; Pysek *et al*. 1995; Rejmanek and Richardson 1996; Theoharides and Dukes 2007; Van Kleunen *et al*. 2010), although some have criticized this approach (Thompson and Davis 2011). Once established, invasive species can also affect the communities that they invade. Clearly, invaders that become the dominant species can significantly change the composition of the communities they invade. However, even when invasives do not form monocultures, they may still have significant, perhaps more subtle, effects. Invaders can alter soil dynamics and resource availability in ways that may benefit some plant species while harming others (Ehrenfeld 2003). The opposite is also true: existing community members can play a role in whether or not invasive species become established in the first place and may limit the abundance of invaders that are able to establish (Levine *et al*. 2004).

There is a long-standing understanding that invasive species can be both a consequence and a cause of changing environments. For example, Vitousek *et al*. (1997) state that ‘Biological invasions are a widespread and significant component of human-caused global environmental change’, while Mack and D'Antonio (1998) state that ‘It is well known that invasions can be promoted by disturbance.’ This has more recently been encapsulated in the driver and passenger models of invasion (MacDougall and Turkington 2005); some invaders are ‘drivers’ of change, while other, non-native ‘passenger’ species merely take the advantage of changing conditions, such as disturbance, to invade a community. It is important to distinguish which model is operating, because management to remove the invader will be unable to mitigate environmental impacts if the invader was not the original change agent (White *et al*. 2013). In some cases, there is support for more than one of these models operating at different times: an invasion can initially take place with the invader being a driver, but later in the process, the invasive is more of an opportunist (White *et al*. 2013).

Once an invader is widespread, a range of subsequent invasion impacts can arise. Jeschke *et al*. (2014) argued for a clearer use of the word ‘impacts’ focusing on clarity about directionality, classification and measurement, ecological and socio-economic changes and scale. It appears that the strongest impacts of plant invasions are seen on plants, both at the species and community levels, rather than on animals or soils (Pyšek *et al*. 2012). Species that are closely related phylogenetically appear to have similar impacts on plant and animal communities (Vilà *et al*. 2015). Some have suggested that the invasion of one species can facilitate the invasion of other non-native species (‘invasional meltdown’), although this has only been clearly documented in a few cases (Simberloff and Von Holle 1999; Simberloff 2011), while other researchers have found evidence for ‘invasional interference’, where invaders may reduce the success or impact of other non-natives (Yang *et al*. 2011; Rauschert and Shea 2012*a*).

However, an important precursor to understanding such complex mechanisms is to document patterns, which are not consistent. Much of the literature has focused on whether or not more diverse communities are more invaded or invasible (Elton 1958; Lonsdale 1999; Stohlgren *et al*. 1999; Naeem *et al*. 2000; Eriksson *et al*. 2006). Various studies have reported both positive (Stohlgren *et al*. 1999) and negative (Hejda *et al*. 2009; Vilà *et al*. 2011, 2015) relationships between native and exotic species richness. These apparently conflicting results have been theorized to be a function of the scale at which the relationship is studied (Shea and Chesson 2002), and may be due to larger-scale studies encompassing more spatial heterogeneity (Davies *et al*. 2005; Sandel and Corbin 2010). In many cases, species identity may be more important than species richness (Crawley *et al*. 1999); this may depend on the functional similarity of the dominant species to the potential invader (Emery 2007; Emery and Gross 2007).

In this study, we address the vegetation patterns associated with two non-native, invasive species. *Carduus nutans* (musk thistle) and *C. acanthoides* (plumeless thistle) are two congeneric, economically important weeds in North and South America, South Africa, Australia and New Zealand (Julien and Griffiths 1999), primarily due to their negative impact in pastures. *Carduus nutans* and *C. acanthoides* are the 2nd and 15th most commonly listed noxious weeds in the USA (Skinner *et al*. 2000). These *Carduus* species have a highly segregated, and relatively stable, distribution in central Pennsylvania with a narrow area of overlap (Allen and Shea 2006; Rauschert *et al*. 2012). To assess their interactions with other plants, we surveyed the vegetation associated with *C. nutans* and *C. acanthoides* by sampling nearly 2000 randomly placed quadrats in four sites of natural thistle co-occurrence in 2 years. We were interested in determining (i) whether there were differences in the composition of the plant community in plots with and without *Carduus*, (ii) whether particular species were associated with the presence of *Carduus* and (iii) whether these associations differed between sites. We hypothesized that, in general, invaded areas would have different plant community associations than non-invaded areas, and we expected that more non-native species would be associated with *Carduus* invasion. We were also interested in whether *C. nutans* was associated with different species than *C. acanthoides*, as a possible mechanism driving the regional spatial segregation of the thistles.

## Methods

### Species description

*Carduus nutans* and *C. acanthoides* are monocarpic perennials of Eurasian origin (Desrochers *et al*. 1988). They are quite similar in appearance, particularly during the rosette stage. Rosettes can occupy a considerable amount of space, with leaves up to 30 cm long (Desrochers *et al*. 1988). Vernalization is required for both species to bolt and flower. Flowering individuals of either species can produce thousands of seeds (McCarty 1982; Feldman and Lewis 1990).

Both species are common in pastures and along roadsides and thrive in disturbed areas (Kok 2001). Establishment of both species depends on the characteristics of potential germination sites, with generally better germination in larger gaps (Panetta and Wardle 1992; Feldman *et al*. 1994; Ruggiero and Shea 2011; Rauschert and Shea 2012*b*). The effects of interspecific competition between the two *Carduus* thistles seem to be similar to the effects of intraspecific competition in an old field setting (Rauschert and Shea 2012*a*).

### Site description

We surveyed the vegetation in four sites of co-occurrence within the narrow area of overlap previously identified in Pennsylvania, USA in 2004 and 2005. Sites were chosen to represent the most common types of invaded areas like pastures, roadsides and abandoned areas, and to contain sufficient (>100) individuals of both species present. In each site, we focused on the few areas of co-occurrence of both species, which led to different sized survey areas in each site. We chose two permanent pastures (PSTR1 and PSTR2), to avoid the tilling and cropping that may break the cycle of biennials and perennials and obscure co-occurrence patterns. Besides regular grazing, both pastures received minimal management consisting of very occasional mowing. PSTR1 (coordinates 40.379N, 77.306W) had mostly *C. acanthoides* present, with a few *C. nutans* individuals; the soil was mostly Calvin shaly silt loam (Soil Survey Staff 2011). We surveyed within an 80 × 30 m area which was used for occasional cattle grazing despite the extremely high density of thistles. In PSTR2 (coordinates 40.225N, 77.431W), we surveyed within two large patches of thistle co-occurrence: an 80 × 25 m section near a temporary stream and a 40 × 45 m section in the centre of the pasture. The soil was mostly Weikert very shaly silt loam (Soil Survey Staff 2011). The managers of both pastures indicated that the thistle infestation was a long-term problem.

The site INDRL (40.183N, 77.238W, soils mostly classified as urban, Soil Survey Staff 2011) was an abandoned industrial site, with the highest densities of *C. nutans* we saw in Pennsylvania. We surveyed within a 40 × 45 m portion of the site containing both species, although the *C. nutans* densities were somewhat lower in that portion of the site.

The site RDG (coordinates 40.301N, 77.400W), located on a ridge along a road, was highly linear: the road was surrounded by a dense forest, and thistles are only found in the cleared area immediately adjacent to the road. According to soil maps, the area consisted mostly of Hazelton extremely stony sandy loam and Dystrochrepts boulder (Soil Survey Staff 2011), but much of the roadside soil appeared to have been brought in with road construction and maintenance. *Carduus nutans* was found more near the top of the slope, whereas *C. acanthoides* was generally found further down the slope. However, a substantial population of *C. acanthoides* was located on an unsurveyed portion of the top of the ridge, indicating that the distribution of the thistles in the survey was not just due to elevational differences. PSTR1, PSTR2 and INDRL were not sprayed with herbicide during this study. Although roadsides are occasionally sprayed in this area, we did not observe signs of herbicide application in the surveyed portions of RDG. Rainfall was high in 2004 (142.6 cm); 2005 (100.4 cm) was much closer to the long-term annual mean for Cumberland County (100.8 cm, The Pennsylvania State Climatologist 2009).

### Field methods

Each site was sampled in both years by placing 1 × 1 m quadrats at random locations throughout the site. Sampling locations were chosen by preselecting random coordinates, in order to avoid problems of periodicity (Krebs 1989); new random locations were chosen each year. Random sampling methods work better than transect methods if there is heterogeneity, although larger numbers of samples may be required to detect rare species (Goslee 2006). A minimum of 10 % of each site was sampled. All plant species present were recorded. Each quadrat was subdivided into nine sectors to allow quantification of within-quadrat species frequency. In addition, in 2005 an abundance estimate was recorded for each species using Daubenmire cover classes (0–5, 5–25, 25–50, 50–75, 75–95, 95–100 %) (Daubenmire 1959; Bonham *et al*. 2004).

### Statistical analysis

As differences in ecological data are often better represented by non-Euclidean distance metrics, we used Non-metric multidimensional scaling (NMDS) to visually explore similarities and differences in composition in thistle versus non-thistle plots and differences between sites. Non-metric multidimensional scaling is an ordination technique that graphs similar plots closer together and dissimilar plots further apart based on ranking distances (Legendre and Legendre 1998). It is more robust than other ordination techniques for analysing community ecological data (Minchin 1987).

To create the distance matrices necessary for NMDS, we constructed three community matrices for each site in each year: (i) presence–absence, (ii) frequencies and (iii) abundance using the midpoint of the cover classes (2005 data only). Distance matrices were calculated using Jaccard distances for presence–absence data, which is suitable for species analyses because it does not consider joint absences, whose meaning is confounded in ecological data (Legendre and Legendre 1998). For the same reason, Bray–Curtis distances were used for frequency and per cent cover data (Legendre and Legendre 1998). *Carduus* thistles were excluded from the community matrix, because we wanted to test for differences among the other community members in plots with and without thistles. Non-metric multidimensional scaling ordination was used to examine clustering of thistle versus non-thistle plots and differences between sites. Non-metric multidimensional scaling ordinations were calculated for one to five dimensions, and principal coordinates ordination was used to establish the starting configuration.

Prior work demonstrated significant autocorrelation in the *Carduus* thistle distribution in these sites (Rauschert *et al*. 2012). We evaluated the spatial pattern in these plots using Mantel correlograms of the community matrix constructed with 10 000 permutations (Legendre and Fortin 1989). The global significance of a correlogram is determined by testing whether at least one correlation coefficient is significant at the *α*′ = *α*/*υ* (Bonferroni corrected level), where *υ* is equal to the number of distance classes; we consider the *α* = 0.05 level (Legendre and Fortin 1989). We used partial Mantel tests with 1000 permutations (Legendre and Fortin 1989) to test for community differences in the thistle and non-thistle plots after spatial structure was accounted for. To test for differences in the communities associated with each of the two thistle species, we subset the data into plots containing only *C. nutans* and those containing only *C. acanthoides*. We used the ‘ecodist’ package version 1.2.9 (Goslee and Urban 2007) in R version 3.1.1 (R Development Core Team 2015) for these analyses.

To identify species that vary between significantly different groups, we performed indicator species analyses, which involve quantifying both the relative abundance as well as whether a species is always present in a group. Our dataset was split into two groups: plots with thistles present or absent (McCune and Grace 2002). Analyses were performed separately in each site in each year. Indicator values range from 100 (perfect indication) to zero, with 25 generally considered to be an acceptable minimum threshold level for a useful indicator species (Dufrene and Legendre 1997). Indicator values were calculated using the ‘indval’ function in the labdsv package in R version 1.4-1 (Roberts 2010). Vector fitting was used with the NMDS ordinations to visualize the effects of indicator species using the ‘vf’ function with 10 000 permutations in the ecodist package (Goslee and Urban 2007).

## Results

**Figure 1.**
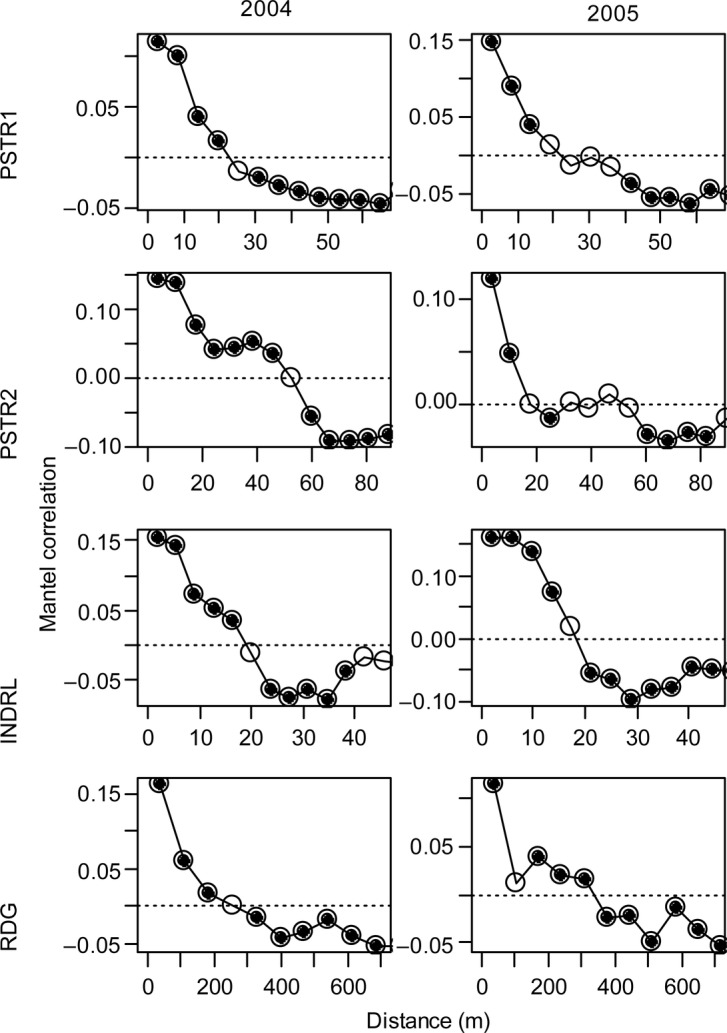
Mantel correlograms using presence–absence data. The correlograms shown are plots of the correlation in the vegetation community at different distance classes. There is a significant positive autocorrelation in all cases, meaning that plots that are closer (in geographical distance) are more likely to be similar. Correlation coefficients that are significantly different from zero are shown with filled dots.

**Figure 2.**
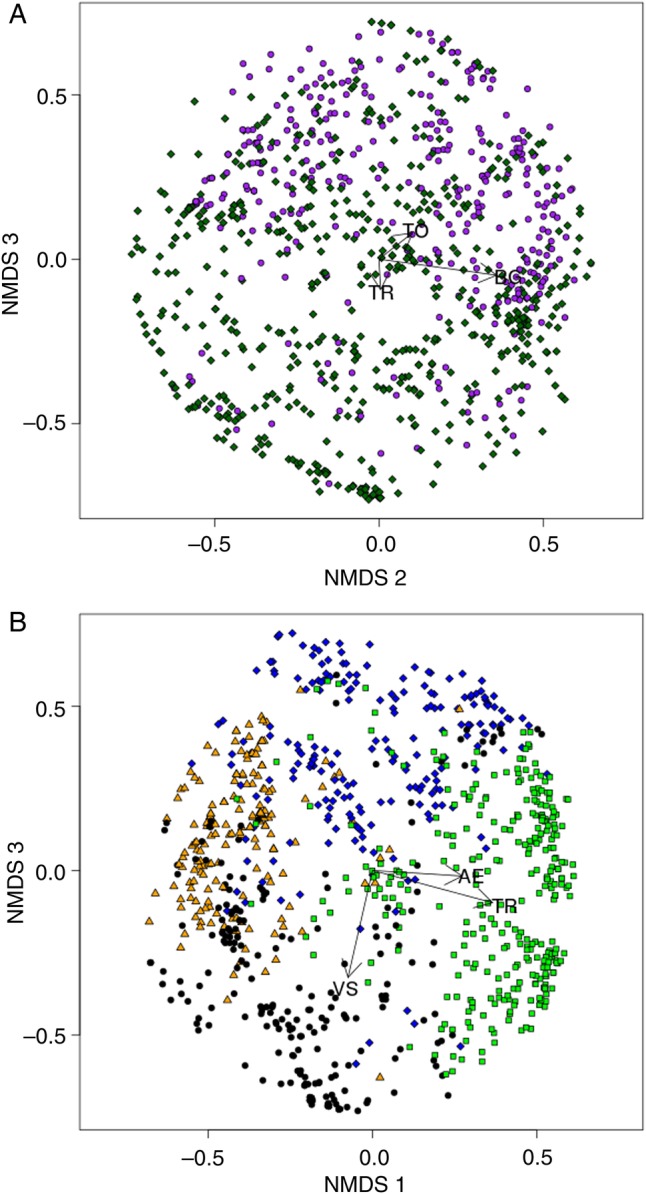
Non-metric multidimensional scaling of per cent cover in all sites for 2005. (A) Plots with thistles are shown in purple and plots without thistles are green. There is a fair degree of clustering of thistle plots. *Trifolium repens* (TR) is associated with thistle absence; *Taraxacum officinale* (TO) and BG are associated with thistle presence. (B) The different sites of study (PSTR1: blue, PSTR2: green, INDRL: yellow, RDG: black). The groups are strongly clustered, with the two pastures also mostly clustered together. *Arrhenatherum elatius* (AE) and TR are associated with PSTR 2. *Vitus* sp. (VS) is associated with RDG. Two axes from the 3D ordinations are displayed to best display the separation between groups.

The proportion of plots that contained *Carduus* thistles varied considerably between sites and somewhat between years: between 14 and 69 % of the plots that we surveyed contained *Carduus* thistles, for a total of 737 plots with and 1173 plots without *Carduus* thistles (Table 1). All sites except RDG had more plots with *C. acanthoides* than *C. nutans* in both years. The Mantel correlograms of the presence–absence data (Fig. 1) revealed the presence of significant spatial structure in the vegetation in these communities. Mantel correlograms of frequency and per cent cover data revealed similar spatial structure (not shown). Plots were generally positively correlated up to distances of around 20–50 m in most sites. The site RDG had significant positive autocorrelation up to ∼300 m in 2005. All correlograms were globally significant. Non-metric multidimensional scaling ordination of per cent cover data pooled from all sites in 2005 (Fig. 2) showed some degree of clustering of thistle plots in terms of per cent cover, as well as strong differences between the sites themselves for both variables. The 3D NMDS solution had substantially lower stress (0.24 instead of 0.35) and higher *r*^2^ (0.57 instead of 0.45) than the 2D solution, and showed clear groupings of sites and vegetation types, so we chose the 3D solution for further analysis.

**Table 1.** Percentages of plots with *Carduus* thistles in the four sites of co-occurrence.

	Site							
	PSTR1		PSTR2		I		R	
	2004	2005	2004	2005	2004	2005	2004	2005
Plots sampled	235	210	324	324	177	180	220	240
Plots with *Carduus* thistles	69 %	64 %	14 %	30 %	60 %	56 %	25 %	15 %
Plots with both species	8 %	3 %	1.2 %	5 %	14 %	12 %	0.5 %	0.4 %
Plots with *C. acanthoides* only	60 %	60 %	10 %	22 %	46 %	43 %	6 %	3 %
Plots with *C. nutans* only	0.4 %	1 %	2 %	4 %	0 %	1 %	19 %	12 %

The partial Mantel tests (Table 2) revealed significant differences in the presence–absence community data in thistle versus non-thistle plots in the site PSTR2 in 2004 and in every site in 2005 except the site RDG. The site RDG did not have significant differences in plot vegetation in any year regardless of the variables examined. The Mantel *r* values were relatively small and indicated that the differences between the community composition in thistle and non-thistle plots were generally not large. The site PSTR2 still had significant differences in the frequencies of the community members in both years, as did the site PSTR1 in 2004 and the site INDRL in 2005. Sites PSTR1, PSTR2 and INDRL were all significantly different in terms of per cent cover plot composition in thistle and non-thistle plots.

**Table 2.** Differences in community in *Carduus* thistle versus non-thistle plots: results of partial Mantel tests. *P ≤ 0.05; **P ≤ 0.01; ***P ≤ 0.001.

Type of data	Site	2004		2005	
		Mantel *r*	*P*-value	Mantel *r*	*P*-value
Presence–absence	PSTR1	0.002	0.456	0.051	0.006**
	PSTR2	0.076	0.013*	0.133	0.001***
	INDRL	−0.005	0.539	0.071	0.001***
	RDG	0.016	0.282	0.003	0.451
Frequency	PSTR1	0.050	0.041*	0.018	0.119
	PSTR2	0.079	0.020*	0.087	0.001**
	INDRL	0.012	0.225	0.096	0.001*
	RDG	−0.017	0.715	0.014	0.290
Per cent cover	PSTR1	–	–	0.053	0.003**
	PSTR2	–	–	0.100	0.001***
	INDRL	–	–	0.098	0.001***
	RDG	–	–	0.013	0.292

**Figure 3.**
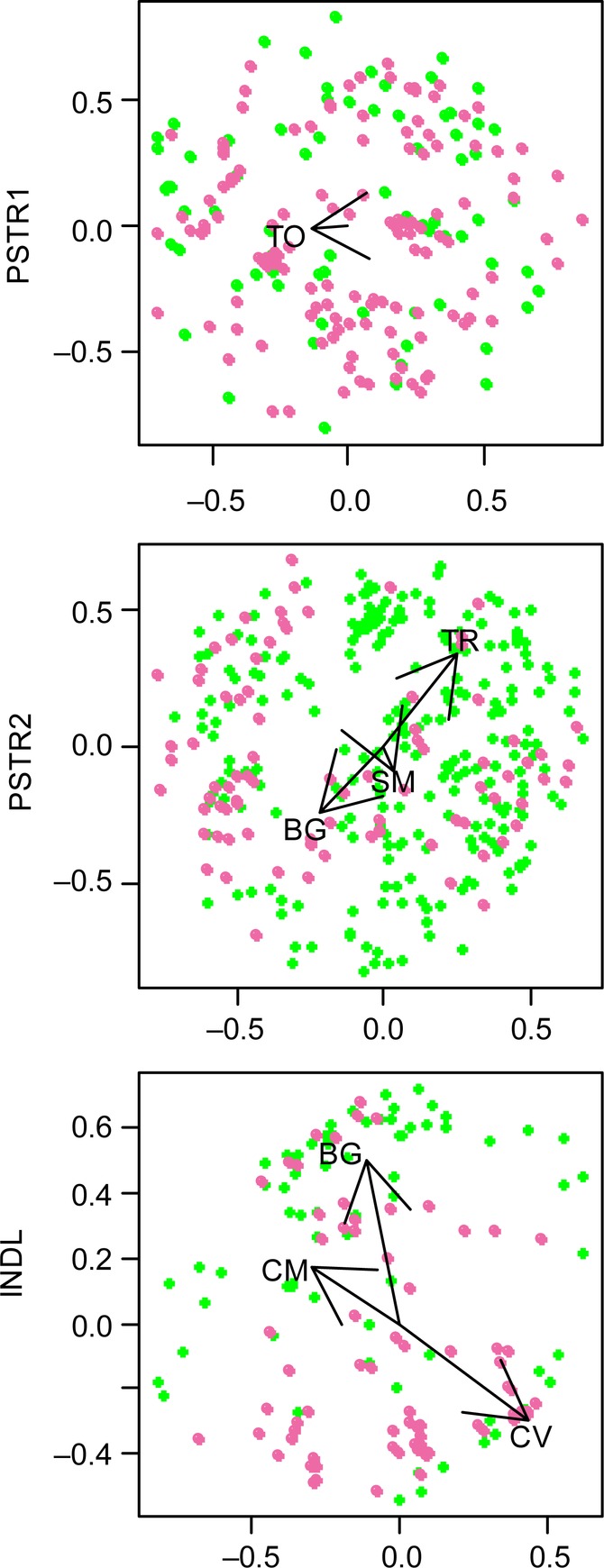
Non-metric multidimensional scaling ordination of per cent cover in three sites. Non-metric multidimensional scaling ordinations are shown for the three sites with significant Mantel *r* results for per cent cover in 2005. Pink dots indicate plots where thistles were present; green dots indicate thistle absence. In PSTR1, TO was associated with thistle presence. In PSTR2, TR was associated with thistle absence; *Sisymbrium officinale* (SM) and BG were associated with thistle presence. In INDRL, *Coronilla varia* (CV) was associated with thistle presence; *Centaurea maculosa* (CM) and BG were associated with thistle absence.

Mostly grasses, such as *Elytrigia repens*, *Arrhenatherum elatius* and *Dactylis glomerata*, and forbs, such as *Plantago* species, *Trifolium* species and *Taraxacum officinale* (TO), were present, with tree seedlings, vines and bushes more common in the site RDG **[see Supporting Information—Appendix S1]**. The native or introduced status of each species was determined using the USDA-Plants Database (USDA-NRCS 2015). Indicator species analyses were performed where partial Mantel tests indicated significant differences in frequency and per cent cover between the vegetation in thistle versus non-thistle plots (Table 3). *Polygonum aviculare* (an introduced species = I), *Trifolium repens* (I), *Centaurea stoebe* (I) and *Chenopodium* sp. were consistently associated with thistle absence (Fig. 3). *Sisymbrium officinale* (I) and *Coronilla varia* (I) were associated with thistle presence. *Taraxacum officinale* (I), *Polygonum persicaria* (I) and bare ground (BG) had mixed associations. *Taraxacum officinale* was associated with thistle presence in PSTR1 in 2005 and with thistle absence in PSTR2 in 2004. *Polygonum persicaria* was associated with thistle presence in PSTR2 in 2004 but thistle absence in PSTR2 2005. Bare ground was associated with thistle presence in PSTR2 in 2005 and with thistle absence in Site INDRL in 2005.

**Table 3.** Results of the indicator species analysis in sites of co-occurrence with significant differences between *Carduus* thistle and non-thistle areas. Indicator values range from 0 to 100 (perfect indications), with 25 as a threshold value for inclusion. Since Site RDG had no significant differences between thistle and non-thistle communities, indicator species analyses were not performed. *P ≤ 0.05; **P ≤ 0.01; ***P ≤ 0.001.

Site	Year	Type of data	Species/category	Group indicated	Indicator value	*P*-value
PSTR1	2004	Frequency	*Chenopodium* sp.	Thistle absence	33	0.001***
			*Polygonum aviculare*	Thistle absence	29	0.001***
PSTR1	2005	Per cent cover	*Taraxacum officinale*	Thistle presence	33	0.018*
PSTR2	2004	Frequency	*Taraxacum officinale*	Thistle absence	30	0.050*
			*Polygonum persicaria*	Thistle presence	41	0.001***
PSTR2	2005	Frequency	*Polygonum persicaria*	Thistle absence	25	0.002**
			Bare ground	Thistle presence	49	0.001***
			*Sisymbrium officinale*	Thistle presence	30	0.001***
PSTR2	2005	Per cent cover	*Trifolium repens*	Thistle absence	54	0.001***
			Bare ground	Thistle presence	52	0.001***
			*Sisymbrium officinale*	Thistle presence	32	0.001***
I	2005	Frequency	Bare ground	Thistle absence	37	0.001***
			*Centaurea stoebe*	Thistle absence	31	0.006**
			*Coronilla varia*	Thistle presence	58	0.001***
I	2005	Per cent cover	Bare ground	Thistle absence	37	0.001***
			*Centaurea stoebe*	Thistle absence	30	0.015*
			*Coronilla varia*	Thistle presence	58	0.001***

We examined whether or not there were differences in plots with *C. nutans*, compared with plots with *C. acanthoides*; however, in PSTR1, PSTR2 and INDRL there were very few plots containing only *C. nutans*. We did not find significant differences between plot types, although the small number of *C. nutans* available for this analysis limits the strength of this conclusion.

## Discussion

Our results highlight the need to examine the invaded plant community when studying invasive species. Many studies focus on the interactions between only a few species and consider the rest of the vegetation to be uniform. The differences that we were able to detect were not immediately obvious when examining these sites, and potentially play a major role in the invasion dynamics if the vegetation is preventing the establishment of thistles in certain areas.

As hypothesized, plots with and without *Carduus* thistles differed in species presence, frequency and cover, except in RDG. There were clear differences in the plant communities of the different sites. The vegetation community in RDG was more that of a forest edge than a field, which may contribute to the lack of differences between thistle and non-thistle plots. It is also possible that the pasture and grassland species more commonly associated with *Carduus* thistles respond differently than forest edge species. In the ordination plot by site (Fig. 2B), it is apparent that the site RDG is strongly separated from the other sites, and one of the most strongly associated species, *Vitus* sp., was not commonly seen in the other fields.

These differences may arise because *Carduus* thistles can be vulnerable to competitive impacts of other species, that is, they may be kept out of areas by biotic resistance. Both of these species are more likely to invade disturbed areas, in part because there is less competition with established vegetation. Their germination and establishment are known to be microsite dependent (Panetta and Wardle 1992; Feldman *et al*. 1994; Ruggiero and Shea 2011; Rauschert and Shea 2012*b*). *Carduus nutans* is particularly sensitive to competition during the rosette stages (Austin *et al*. 1985), and is vulnerable to allelopathic effects of other species (Wardle *et al*. 1995). Increasing competition with other species has been suggested as a possible management option (Kok *et al*. 1986; Wardle *et al*. 1995). Jongejans *et al*. (2007) found that mowing the surrounding vegetation influenced whether or not *C. acanthoides* could invade; frequent mowing lead to dense, lawn-like vegetation which was not conducive to *C. acanthoides* establishment.

The community differences observed may also be because *C. nutans* and *C. acanthoides* are influencing the surrounding vegetation. In a global analysis of plant invasion, European grassland species, including *Carduus nutans*, were found to be highly successful invaders, more so than grassland species from other parts of the world (Hejda *et al*. 2015). Both species have been reported as having allelopathic effects on other species (Woodward and Glenn 1983; Wardle *et al*. 1991). In particular, *C. nutans* may interfere with the nitrogen-fixing abilities of *T. repens* through allelopathic effects of decaying rosette leaves; this may lead to lower nitrogen availability (Wardle *et al*. 1994). It has also been suggested that *C. nutans*’ allelopathic effects may alter the outcome of competitive interactions between grasses and legumes to favour grasses (Wardle *et al*. 1994). The allelochemical most likely responsible for these effects in *C. acanthoides* was recently identified as aplotaxene (Silva *et al*. 2014).

The species identity of immediate neighbours may play a large role in the community dynamics. Of the several species consistently associated with thistle absence, *T. repens* (an introduced species) is known to both be affected by and to affect *C. nutans* (Wardle *et al*. 1992, 1993, 1994). This is particularly important as *T. repens* is a desirable species in pastures, compared with many of the other species we encountered, which are also undesirable pest species. *Centaurea stoebe* is another invasive species with a similar growth habit as *Carduus* thistles. Although its interactions with *Carduus* thistles have not been explicitly studied, it is also believed to be allelopathic (Bais *et al*. 2003). Consistent with what we found in this study, *P. aviculare* (I) is known to be associated with different microhabitats than *C. acanthoides* (Milton *et al*. 1997).

The association of potentially strong non-native competitors *S. officinale* and *C. varia* with thistle presence was somewhat surprising. The positive association with *C. varia* occurred in INDRL, which is an abandoned industrial area of varying soil fertility. The association is most likely driven by both species only being able to grow in certain parts of the site.

Several species had mixed associations with thistle presence. *Polygonum persicaria* is considered an introduced facultative wetland species in Pennsylvania (USDA-NRCS 2015) and in PSTR2 was most frequently found growing along a temporary stream. It is possible that in 2005, areas which had supported *P. persicaria* as well as *Carduus* thistles in 2004 were too dry for *P. persicaria.* Note that given the spatial structure detected in the quadrat composition in general, it is also possible that associations or disassociations observed are in response to other heterogeneities in the site rather than directly to other plant species. Interestingly, most grasses, which are typically desirable pasture species, were not associated with thistle presence or absence, thus they are presumably not harmed by thistle presence.

Invasive species are often viewed as having a different effect than residents, even if they are naturalized, because they have no shared evolutionary history with the species in their invaded ranges. However, the plant species significantly associated with *Carduus* thistles were all non-native species. Interestingly, several of the species that we found are also associated with these *Carduus* thistles in their native ranges. Doing *et al*. (1969) list other common members of the *C. nutans* and *C. acanthoides* group (Onopordion communities) in their native ranges; many of the species listed were also found in our sites (*Meliolotus albus*, *M. officinalis*, *Verbascum thapsus*, *Datura stramonium*, *Cirsium arvense*, *Achillea millefolium*), although the dominant species appear to be different. Thus presumably a large number of these species are not co-occurring for the first time in central Pennsylvania, and many of them have likely been living there together for several centuries. This observation is consistent with biotic homogenization, or an increase in the similarity of communities worldwide, which is mainly driven by the invasion of the same species (McKinney and Lockwood 1999; Baiser *et al*. 2012). In the case of communities already heavily invaded by non-native species, the interactions between non-native species may lead to invasional interference by resident non-natives, perhaps limiting further detrimental effects (Rauschert and Shea 2012*a*).

In fact, all sites examined fit the description of ‘novel ecosystems’ in that they have experienced and will continue to experience heavy human impacts including physical disturbance and the introduction of non-native species (Hobbs *et al*. 2006). If a shared evolutionary history may allow species to adapt to each other's competitive strategies, it may be that members of the same original communities are best suited to co-occur in these new areas. Thus, some of these non-native species may be drivers, while others may be passengers, and the only way to distinguish between them will be via experimentation.

When examining a current invasion, it can be difficult to disentangle the initial biotic resistance of a community from the subsequent impacts of an invasion (Bennett *et al*. 2014). Because conditions change during invasion itself, it is possible that invaded communities initially had characteristics associated with biotic resistance, including higher diversity (Bennett *et al*. 2014). In addition, sometimes conditions change for other reasons, and invasives were just ‘passengers’ of the change (MacDougall and Turkington 2005). Experimental manipulation is required to disentangle these factors.

## Conclusions

Our research takes the first critical step towards describing the relationship between these invasive thistles and the invaded vegetation. We clearly showed differences in the plant community associations in areas with and without these two species. Some species had consistent associations with *Carduus* thistle absence (e.g. *T. repens*, *C. stoebe*), others were associated with *Carduus* thistles (e.g. *C. varia*) and others had mixed associations. The next step will be to clarify the direction of causality: do the invaders establish and grow best in these habitats, or do they alter the vegetation once they arrive or both? Future research now needs to elucidate the mechanisms underlying these results.

## Sources of Funding

This work was partially supported by United States Department of Agriculture Cooperative State Research, Education, and Extension Service National Research Initiative (Biology of Weedy and Invasive Plants) grant #2002-35320-1228 to K.S. and a National Space and Aeronautics Administration Space Grant Fellowship to E.S.J.R.

## Contributions by the Authors

E.S.J.R. planned the study, executed the field work and analyses, and wrote and edited the paper. K.S. planned the study and contributed to the writing and editing of the manuscript. S.G. helped design and execute the analyses and substantially revised the manuscript.

## Conflict of Interest Statement

None declared.

## Supporting Information

The following additional information is available in the online version of this article –

**Appendix S1.** List of the plant species found in four fields of co-occurrence and their native/introduced status.

Additional Information
