# Supplementary material for: Plant community associations of two invasive thistles
Source: AoB Plants. 2015 Jun 2;7:plv065. doi: 10.1093/aobpla/plv065 (PMC4571105; doi:10.1093/aobpla/plv065)
Supplement: Additional Information [file supp_7_plv065_index.html]

Plant community associations of two invasive thistles — Plant community associations of two invasive thistles — Additional Information 

# Plant community associations of two invasive thistles

## Additional Information

Additional Information

- Additional Information - Docx file
