# Supplementary material for: Plant community associations of two invasive thistles
Source: AoB Plants. 2015 Jun 2;7:plv065. doi: 10.1093/aobpla/plv065 (PMC4571105; doi:10.1093/aobpla/plv065)
Supplement: Additional Information [file supp_plv065_plv065supp.docx]

| Appendix 1: Plant species identified in four sites of *Carduus* thistle co-occurrence  Native status is indicated by N=native, I=introduced, U = unknown.   \| **Plants** \| \| Present in \| \| \| \| \| \| \|  \| \| Present in \| \| \| \| \| \| \| \| \| --- \| --- \| --- \| --- \| --- \| --- \| --- \| --- \| --- \| --- \| --- \| --- \| --- \| --- \| --- \| --- \| --- \| --- \| --- \| \| ***Scientific name*** \| \| ***PSTR*** \| \| \| ***I*** \| \| ***R*** \| \| ***Scientific name*** \| \| ***PSTR*** \| \| \| \| ***I*** \| \| ***R*** \| \| \|  \| \| ***1*** \| ***2*** \| \|  \| \|  \| \|  \| \| ***1*** \| \| ***2*** \| \|  \| \|  \| \| \| *Acer rubrum* (N) \| \|  \| *x* \| \|  \| \| *x* \| \| *Phytolacca americana* (N) \| \| *x* \| \|  \| \|  \| \|  \| \| \| *Ailanthus altissima* (I) \| \|  \|  \| \|  \| \| *x* \| \| *Plantago lanceolata* (I) \| \| *x* \| \| *x* \| \| *x* \| \| *x* \| \| \| *Alliaria petiolata* (I) \| \|  \|  \| \|  \| \| *x* \| \| *Plantago major* (I) \| \| *x* \| \| *x* \| \| *x* \| \| *x* \| \| \| *Allium canadense* (N) \| \| *x* \|  \| \| *x* \| \|  \| \| *Polygonum aviculare* (I) \| \| *x* \| \|  \| \|  \| \|  \| \| \| *Allium vineale* (I) \| \| *x* \|  \| \|  \| \| *x* \| \| *Polygonum cuspidatum* (I) \| \|  \| \|  \| \|  \| \| *x* \| \| \| *Arctium minus* (I) \| \| *x* \| *x* \| \|  \| \| *x* \| \| *Polygonum persicaria* (I) \| \| *x* \| \| *x* \| \|  \| \| *x* \| \| \| *Arrhenatherum elatius* (I) \| \| *x* \| *x* \| \| *x* \| \|  \| \| *Portulaca oleracea* (I) \| \| *x* \| \| *x* \| \| *x* \| \|  \| \| \| *Asclepias syriaca* (N) \| \| *x* \|  \| \|  \| \| *x* \| \| *Potentilla recta* (I) \| \| *x* \| \| *x* \| \|  \| \|  \| \| \| *Barbarea vulgaris* (I) \| \| *x* \|  \| \|  \| \|  \| \| *Prunus* species (U) \| \|  \| \|  \| \|  \| \| *x* \| \| \| *Capsella bursa-pastoris* (I) \| \|  \| *x* \| \|  \| \|  \| \| *Quercus* species (U) \| \|  \| \|  \| \|  \| \| *x* \| \| \| *Carduus acanthoides* (I) \| \| *x* \| *x* \| \| *x* \| \| *x* \| \| *Rhus* species (U) \| \|  \| \|  \| \|  \| \| *x* \| \| \| *Carduus nutans* (I) \| \| *x* \| *x* \| \| *x* \| \| *x* \| \| *Robinia pseudoacacia* (N) \| \|  \| \|  \| \|  \| \| *x* \| \| \| *Carex lurida* (N) \| \|  \| *x* \| \|  \| \|  \| \| *Rorippa sylvestris* (I) \| \|  \| \|  \| \|  \| \| *x* \| \| \| *Centaurea stoebe* (I) \| \| *x* \|  \| \| *x* \| \| *x* \| \| *Rosa multiflora* (I) \| \|  \| \|  \| \|  \| \| *x* \| \| \| *Chenopodium album* (I) \| \| *x* \| *x* \| \|  \| \|  \| \| *Rosa* species (U) \| \|  \| \| *x* \| \|  \| \|  \| \| \| *Chenopodium* species (U) \| \| *x* \|  \| \|  \| \|  \| \| *Rubus* species (U) \| \|  \| \|  \| \| *x* \| \| *x* \| \| \| *Cirsium arvense* (I) \| \| *x* \| *x* \| \| *x* \| \| *x* \| \| *Rumex crispus* (I) \| \| *x* \| \|  \| \|  \| \| *x* \| \| \| *Cirsium vulgare* (I) \| \| *x* \| *x* \| \| *x* \| \| *x* \| \| *Schedonorus arundinaceus* (I) \| \| *x* \| \|  \| \|  \| \|  \| \| \| *Commelina communis* (I) \| \|  \|  \| \|  \| \| *x* \| \| *Securigaria varia* (I) \| \| *x* \| \|  \| \| *x* \| \| *x* \| \| \| *Dactylis glomerata* (I) \| \| *x* \| *x* \| \|  \| \|  \| \| *Sisymbrium officinale* (I) \| \|  \| \| *x* \| \|  \| \|  \| \| \| *Datura stramonium* (I) \| \|  \| *x* \| \|  \| \|  \| \| *Solidago* species (N) \| \| *x* \| \|  \| \| *x* \| \| *x* \| \| \| *Daucus carota* (I) \| \| *x* \| *x* \| \| *x* \| \| *x* \| \| *Stellaria media* (I) \| \| *x* \| \| *x* \| \|  \| \|  \| \| \| *Dipsacus fullonum* (I) \| \| *x* \| *x* \| \| *x* \| \| *x* \| \| *Taraxacum officinale* (I,N) \| \| *x* \| \| *x* \| \| *x* \| \| *x* \| \| \| *Elymus repens* (I) \| \| *x* \| *x* \| \| *x* \| \| *x* \| \| *Thlaspi arvense* (I) \| \| *x* \| \|  \| \| *x* \| \|  \| \| \| *Erigeron philadelphicus* (N) \| \| *x* \| *x* \| \| *x* \| \| *x* \| \| *Toxicodendron radicans* (N) \| \|  \| \|  \| \|  \| \| *x* \| \| \| *Galium* species (U) \| \| *x* \|  \| \| *x* \| \|  \| \| *Tragopogon pratensis* (I) \| \|  \| \|  \| \|  \| \| *x* \| \| \| *Glechoma hederacea* (I) \| \| *x* \|  \| \| *x* \| \|  \| \| *Trifolium arvense* (I) \| \| *x* \| \|  \| \|  \| \|  \| \| \| *Hesperis matronalis* (I) \| \|  \|  \| \| *x* \| \| *x* \| \| *Trifolium pretense* (I) \| \| *x* \| \| *x* \| \| *x* \| \|  \| \| \| *Impatiens pallida* (N) \| \|  \|  \| \|  \| \| *x* \| \| *Trifolium repens* (I) \| \| *x* \| \| *x* \| \| *x* \| \|  \| \| \| *Juncus effusus var. solutus* (N) \| \|  \| *x* \| \|  \| \|  \| \| *Verbascum thapsus* (I) \| \| *x* \| \|  \| \|  \| \|  \| \| \| *Lepidium campestre* (I) \| \| *x* \|  \| \|  \| \|  \| \| *Veronica officinalis* (I) \| \| *x* \| \|  \| \|  \| \|  \| \| \| *Leucanthemum vulgare* (I) \| \|  \|  \| \|  \| \| *x* \| \| *Vinca minor* (I) \| \|  \| \|  \| \|  \| \| *x* \| \| \| *Linaria vulgaris* (I) \| \| *x* \|  \| \| *x* \| \| *x* \| \| *Vitus* species (U) \| \| *x* \| \|  \| \|  \| \| *x* \| \| \| *Liriodendron tulipifera* (N) \| \|  \|  \| \|  \| \| *x* \| \| *Zea mays* (I) \| \| *x* \| \|  \| \|  \| \|  \| \| \| *Lonicera* species (U) \| \|  \|  \| \|  \| \| *x* \| \| Unknown moss (U) \| \|  \| \|  \| \| *x* \| \|  \| \| \| *Malva neglecta* (I) \| \| *x* \| *x* \| \|  \| \|  \| \| Unknown dicot (U) \| \| *x* \| \| *x* \| \| *x* \| \| *x* \| \| \| *Medicago lupulina* (I) \| \|  \| *x* \| \| *x* \| \|  \| \| Unknown grass (U) \| \| *x* \| \| *x* \| \| *x* \| \| *x* \| \| \| *Melilotus alba* (I) \| \| *x* \|  \| \| *x* \| \| *x* \| \|  \| \|  \| \|  \| \|  \| \|  \| \| \| *Melilotus officinalis* (I) \| \| *x* \| *x* \| \| *x* \| \| *x* \| \| **Abiotic categories** \| \|  \| \|  \| \|  \| \|  \| \| \| *Nepeta cataria* (N) \| \| *x* \| *x* \| \| *x* \| \| *x* \| \| Bare ground \| \| *x* \| \| *x* \| \| *x* \| \| *x* \| \| \| *Oxalis strica* (I) \| \| *x* \| *x* \| \| *x* \| \|  \| \| Water \| \|  \| \| *x* \| \| *x* \| \|  \| \| \| *Parthenocissus quinquefolia* (N) \| \| *x* \|  \| \| *x* \| \| *x* \| \| Rock \| \|  \| \|  \| \| *x* \| \| x \| \| \| *Phleum pretense* (I) \| \| *x* \| *x* \| \| *x* \| \|  \| \|  \| \|  \| \|  \| \|  \| \|  \| \| \|  \|  \| \| \|  \| \|  \| \|  \| \|  \| \|  \| \|  \| \|  \| \|  \| \| \|  \|  \| \| \|  \| \|  \| \|  \| \|  \| \|  \| \|  \| \|  \| \|  \| \| |
| --- | --- | --- | --- | --- | --- | --- | --- | --- | --- | --- | --- | --- | --- | --- | --- | --- | --- | --- | --- | --- | --- | --- | --- | --- | --- | --- | --- | --- | --- | --- | --- | --- | --- | --- | --- | --- | --- | --- | --- | --- | --- | --- | --- | --- | --- | --- | --- | --- | --- | --- | --- | --- | --- | --- | --- | --- | --- | --- | --- | --- | --- | --- | --- | --- | --- | --- | --- | --- | --- | --- | --- | --- | --- | --- | --- | --- | --- | --- | --- | --- | --- | --- | --- | --- | --- | --- | --- | --- | --- | --- | --- | --- | --- | --- | --- | --- | --- | --- | --- | --- | --- | --- | --- | --- | --- | --- | --- | --- | --- | --- | --- | --- | --- | --- | --- | --- | --- | --- | --- | --- | --- | --- | --- | --- | --- | --- | --- | --- | --- | --- | --- | --- | --- | --- | --- | --- | --- | --- | --- | --- | --- | --- | --- | --- | --- | --- | --- | --- | --- | --- | --- | --- | --- | --- | --- | --- | --- | --- | --- | --- | --- | --- | --- | --- | --- | --- | --- | --- | --- | --- | --- | --- | --- | --- | --- | --- | --- | --- | --- | --- | --- | --- | --- | --- | --- | --- | --- | --- | --- | --- | --- | --- | --- | --- | --- | --- | --- | --- | --- | --- | --- | --- | --- | --- | --- | --- | --- | --- | --- | --- | --- | --- | --- | --- | --- | --- | --- | --- | --- | --- | --- | --- | --- | --- | --- | --- | --- | --- | --- | --- | --- | --- | --- | --- | --- | --- | --- | --- | --- | --- | --- | --- | --- | --- | --- | --- | --- | --- | --- | --- | --- | --- | --- | --- | --- | --- | --- | --- | --- | --- | --- | --- | --- | --- | --- | --- | --- | --- | --- | --- | --- | --- | --- | --- | --- | --- | --- | --- | --- | --- | --- | --- | --- | --- | --- | --- | --- | --- | --- | --- | --- | --- | --- | --- | --- | --- | --- | --- | --- | --- | --- | --- | --- | --- | --- | --- | --- | --- | --- | --- | --- | --- | --- | --- | --- | --- | --- | --- | --- | --- | --- | --- | --- | --- | --- | --- | --- | --- | --- | --- | --- | --- | --- | --- | --- | --- | --- | --- | --- | --- | --- | --- | --- | --- | --- | --- | --- | --- | --- | --- | --- | --- | --- | --- | --- | --- | --- | --- | --- | --- | --- | --- | --- | --- | --- | --- | --- | --- | --- | --- | --- | --- | --- | --- | --- | --- | --- | --- | --- | --- | --- | --- | --- | --- | --- | --- | --- | --- | --- | --- | --- | --- | --- | --- | --- | --- | --- | --- | --- | --- | --- | --- | --- | --- | --- | --- | --- | --- | --- | --- | --- | --- | --- | --- | --- | --- | --- | --- | --- | --- | --- | --- | --- | --- | --- | --- | --- | --- | --- | --- | --- | --- | --- | --- | --- | --- | --- | --- | --- | --- | --- | --- | --- | --- | --- | --- | --- | --- | --- | --- | --- | --- | --- | --- | --- | --- | --- | --- | --- | --- | --- | --- | --- | --- | --- | --- | --- | --- | --- | --- | --- | --- | --- | --- | --- | --- | --- | --- | --- | --- | --- | --- | --- | --- | --- | --- | --- | --- | --- | --- | --- | --- | --- | --- | --- | --- | --- | --- | --- | --- | --- | --- | --- | --- | --- | --- | --- | --- | --- | --- | --- | --- | --- | --- | --- | --- | --- | --- | --- | --- | --- | --- | --- | --- | --- | --- | --- | --- | --- | --- | --- | --- | --- | --- | --- | --- | --- | --- | --- | --- | --- | --- | --- | --- | --- | --- | --- | --- | --- | --- | --- | --- | --- | --- | --- | --- | --- | --- | --- | --- | --- | --- | --- | --- | --- | --- | --- | --- | --- | --- | --- | --- | --- | --- | --- | --- | --- | --- | --- | --- | --- | --- | --- | --- | --- | --- | --- | --- | --- | --- | --- | --- | --- | --- | --- | --- | --- | --- | --- | --- | --- | --- | --- | --- | --- | --- | --- | --- | --- | --- | --- | --- | --- | --- | --- | --- | --- | --- | --- | --- | --- | --- | --- | --- | --- | --- | --- | --- | --- | --- | --- | --- | --- | --- | --- | --- | --- | --- | --- | --- | --- | --- | --- | --- | --- | --- | --- | --- | --- | --- | --- | --- | --- | --- | --- | --- | --- | --- | --- | --- | --- | --- | --- | --- | --- | --- | --- | --- | --- | --- | --- | --- | --- | --- | --- | --- | --- | --- | --- | --- | --- | --- | --- | --- | --- | --- | --- | --- | --- | --- | --- | --- | --- | --- | --- | --- | --- | --- | --- | --- | --- | --- | --- | --- | --- | --- | --- | --- | --- | --- | --- | --- | --- | --- | --- | --- | --- | --- | --- | --- | --- | --- | --- | --- | --- | --- | --- | --- | --- | --- | --- | --- | --- | --- | --- | --- | --- | --- | --- | --- | --- | --- | --- | --- | --- | --- | --- | --- | --- | --- | --- | --- | --- | --- | --- | --- | --- | --- | --- | --- | --- | --- | --- | --- | --- | --- | --- | --- | --- | --- | --- | --- | --- | --- | --- | --- | --- | --- | --- | --- | --- | --- | --- | --- | --- | --- | --- | --- | --- | --- | --- | --- | --- | --- | --- | --- | --- | --- | --- | --- | --- | --- | --- | --- | --- | --- | --- | --- | --- | --- | --- | --- | --- | --- | --- | --- | --- | --- | --- | --- | --- | --- | --- | --- | --- | --- | --- | --- | --- | --- | --- | --- | --- | --- | --- | --- | --- | --- | --- | --- | --- | --- | --- | --- | --- | --- | --- | --- | --- | --- | --- | --- | --- | --- | --- | --- | --- | --- | --- | --- | --- | --- | --- | --- | --- | --- | --- | --- | --- | --- | --- | --- | --- | --- | --- | --- | --- | --- | --- | --- | --- | --- | --- | --- | --- | --- | --- | --- | --- | --- | --- | --- | --- | --- | --- | --- | --- | --- | --- | --- | --- | --- | --- | --- | --- | --- | --- | --- | --- | --- | --- | --- | --- | --- |
